# Polyurethane Nanocomposite Coatings Coupled with Titanium-Based Conversion Layers for Enhanced Anticorrosion, Icephobic Properties, and Surface Protection

**DOI:** 10.3390/molecules29163901

**Published:** 2024-08-17

**Authors:** Shamim Roshan, Reza Jafari, Gelareh Momen

**Affiliations:** Department of Applied Sciences, University of Québec in Chicoutimi (UQAC), 555, Boul. de l’Université, Chicoutimi, QC G7H 2B1, Canada; gelareh_momen@uqac.ca

**Keywords:** polyurethane, titanium, cerium, conversion coating, icephobicity, corrosion, superhydrophobicity

## Abstract

This study examines the efficacy of icephobic polyurethane nanocomposite coatings in mitigating corrosion on an aluminum substrate. A titanium-based conversion coating is applied to modify the substrate, and the research focuses on optimizing the dual functionalities of icephobicity and anticorrosion within the polyurethane coatings while ensuring strong substrate adhesion. The coatings are formulated using fluoropolyol, isocyanate, and silica nanoparticles treated with polydimethylsiloxane. Surface properties are analyzed using contact angles, contact angle hysteresis measurements, and atomic force microscopy, and the coatings’ icephobicity is evaluated through differential scanning calorimetry, freezing time delay, ice adhesion under impact and non-impact conditions, and ice accretion tests. The corrosion resistance and adhesive strength of the coatings are assessed using electrochemical impedance spectroscopy and cross-cut tests, respectively. Increasing the concentration of silica nanoparticles to 10 wt.% increases contact angles to 167°, although the 4 wt.% coating produces the lowest contact angle hysteresis (3° ± 0.5°) and ice nucleation temperature (−23 °C). The latter coating is then applied to a substrate pretreated with a titanium/cerium-based conversion coating. This prepared surface maintains an ice adhesion of about 15 kPa after 15 icing/de-icing cycles and provides approximately 90 days of surface protection (|Z|_lf_ = 1.6 × 10^9^ Ω·cm^2^). Notably, the impedance value exceeds that of untreated substrates, underscoring the effectiveness of the titanium/cerium-based conversion coating in enhancing both corrosion resistance and coating adhesion to the substrate.

## 1. Introduction

Organic coatings are commonly used to protect metal structures in industry [1,2]; however, these coatings do not provide complete barrier protection against water, oxygen, and corrosive substances [3,4,5]. Producing a water-repellent coating with improved barrier properties can be challenging. The anticorrosion resistance and barrier performance of organic coatings are influenced by various factors, e.g., unfilled/filled pores, open/blocked diffusion paths, a variety of coating defects, the length of the penetration path for corrosive ingredients, the formation of cracks, and the surface free energy of the coating [6,7]. Recently, there has been extensive research on the preparation of hydrophobic and superhydrophobic coatings, as these coatings have the ability to repel water or slow water penetration [8,9,10,11,12,13]. Moreover, a strong correlation has been found between hydrophobicity and reduced ice adhesion [14,15]. Superhydrophobic surfaces (i.e., those with a water contact angle (WCA) > 150°) have demonstrated promising anti-icing performance [16].

Polyurethane polymers are widely used organic coatings in industry [17,18]. Moreover, adding nanoparticles to polyurethane coatings can improve a coating’s mechanical, physical, and barrier properties and alter its surface characteristics [19,20]. Various types of nanoparticles, such as clay [21], graphene [22,23], carbon nanotubes [24], and silica [25,26,27], have been used to enhance the hydrophobicity and anticorrosion performance of organic coatings. Silica nanoparticles are particularly favored because of their high mechanical strength, thermal and chemical stability, commercial availability, and ease of use for modifying surfaces [28,29,30,31]. The wettability of a coating is strongly influenced by the addition and distribution of silica nanoparticles to polymer-based coatings [32,33].

Przybyszewski et al. developed polyurethane (PU) nanocomposite coatings modified with nanosilica to enhance the coatings’ hydrophobic and anti-icing properties. The polyurethane nanocomposite coatings—enhanced by treating nanosilica with bis(trimethylsilyl)amine (C_6_H_19_NSi_2_) and silane-based compounds—improved hydrophobicity (up to 114° for static water contact angles) and anti-icing properties relative to a non-modified PUR topcoat [34]. Lei et al. produced a durable superhydrophobic coating with excellent anti-icing properties that is suitable for harsh environmental conditions, such as those in polar regions. The researchers achieved these properties by integrating almost 50 wt.% (based on resin and hardener) nanosilica modified with fluoroalkyl silane and aminosilane into a polyurethane matrix through a spraying and curing process. The resulting coating exhibited a high WCA (162°) and a low sliding angle (SA), testifying to its strong superhydrophobicity. These properties remained robust even after rigorous mechanical testing, including 200 cycles of sandpaper abrasion. Furthermore, the coating delayed ice formation from 29 s on a metallic substrate to 750 s on the coating and reduced ice adhesion strength over multiple icing/de-icing cycles [35].

Wang et al. [36] found that increasing the nanoparticle content enhanced the hydrophobicity of a coating and thus reduced the ice adhesion strength. However, beyond a certain threshold (40 wt.% particle content), cracks caused by higher particle loading started to increase the shear stress required for ice removal, demonstrating an ice anchoring effect. The study emphasized the need for an optimal nanoparticle content that maximizes hydrophobicity yet minimizes crack formation to produce durable and effective icephobic surfaces. Incorporating both hydrophobicity and anticorrosion features in a single-layer coating poses significant challenges because enhancing hydrophobic properties generally requires a relatively high nanoparticle content to alter the surface roughness, and cracks can form within the coating at these higher concentrations.

Ejenstam et al. explored the use of varying amounts of hydrophobic fumed silica in a crosslinked polymer matrix of polydimethylsiloxane with fluoro-based materials. They found that whereas the nanosilica effectively slowed water permeation through the coating, higher concentrations of nanosilica produced deep and large cracks [37]. High concentrations of nanoparticles not only create cracks in the coatings but also decrease the adhesion of the metal/coating interface because of the lack of polymers [20]. Surface pretreatment of metals can enhance a coating’s adhesion to the substrate. Conversion coatings create a dense and well-bonded barrier layer on metal surfaces through a chemical reaction between the metal and specific acid or salt solutions [38]. These coatings have been improved to enhance the metal substrate’s resistance to corrosion and strengthen the adhesion between the metal and organic coatings [39,40]. In our recent study, we found that a titanium-based conversion coating modified with cerium ions significantly increased the adhesion strength and anticorrosion performance of a polyurethane coating because of the uniform and rough structure of the titanium/cerium-based conversion coating [41]. Therefore, this type of pretreatment is effective at improving the durability of the final coatings.

To our knowledge, no study has explored enhancing the durability of multifunctional polyurethane nanocomposites with a focus on anticorrosion and icephobic applications using titanium/cerium conversion coatings (Ti/Ce-CC). The fabrication of these coatings is both fast and cost-effective and involves applying a H_2_TiF_6_-based conversion coating of optimal pH, concentration, and immersion duration to a 2024T3 aluminum alloy via immersion methods. A two-component fluoropolyol and isocyanate-based solution containing 4 wt.% polydimethylsiloxane-modified silica nanoparticles is then sprayed onto the pretreated substrate. The designed coating integrates icephobicity, superhydrophobicity, and enhanced anti-corrosion performance exceeding three months in a 3.5 wt.% NaCl solution. Achieving prolonged anti-corrosion performance often poses a challenge due to the high nanoparticle concentration employed in most research. We examined the icephobic characteristics of the superhydrophobic coating and assessed the impact of a thin layer of pretreatment coating on the durability of this novel icephobic and corrosion-resistant coating. Notably, these properties remained intact after multiple anti-icing/de-icing cycles and extended exposure to corrosive environments.

## 2. Results and Discussion

### 2.1. Surface Characterization

To evaluate the hydrophobic properties of the prepared nanocomposite coatings, we first investigated the effect of varying concentrations of nanosilica on coating wettability. The measured contact angle and contact angle hysteresis values of all samples are listed in Table 1. The CA of the thermoset polyurethane without hydrophobic nanosilica was 91°. Adding 4 wt.% of hydrophobic nanosilica shifted the wettability to superhydrophobic. This 4 wt.% concentration allowed the nanoparticles to successfully migrate to the surface, reducing the surface’s free energy (as the SiO_2_ is modified with polydimethylsiloxane [12]) and altering its roughness (and thus increasing the WCA), in turn affecting the surface wettability. Moreover, the lower surface energy of silica nanoparticles relative to that of the polyurethane coating facilitated their migration to the surface, as did the solvent’s adequate evaporation rate [20]. The WCA for FPU-SiO_2_ 7% and FPU-SiO_2_ 10% were 165° and 169°, respectively, slightly higher than that for FPU-SiO_2_ 4%. Advancing and receding CAs less than 10° demonstrate lotus-type hydrophobicity in which air is trapped within the surface pores to allow water droplets to easily roll off [42]. Nevertheless, the FPU-SiO_2_ 4% produced the lowest CAH. Samples with more than 4 wt.% nanoparticles showed lower receding angles and higher advancing angles.

**Figure 1:**
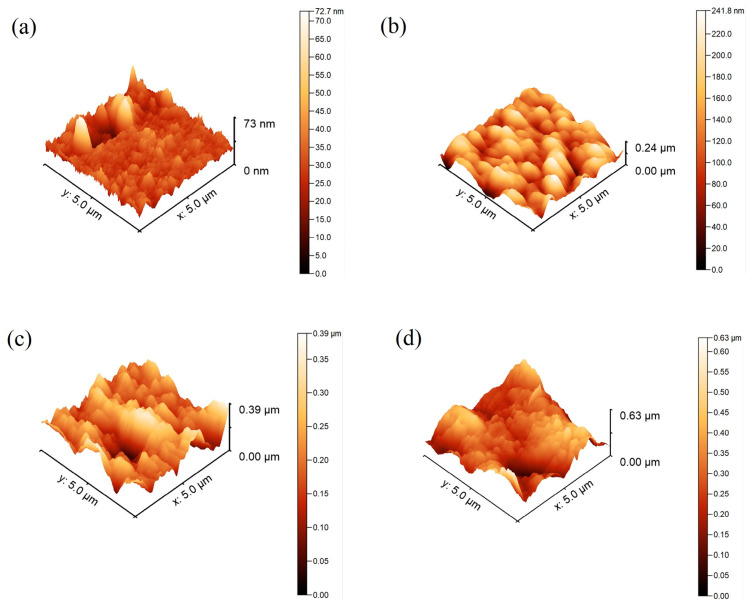
Atomic force microscopy (AFM) images of the coatings’ topography: (**a**) FPU, (**b**) FPU-SiO_2_ 4%, (**c**) FPU-SiO_2_ 7%, and (**d**) FPU-SiO_2_ 10%.

The topography of an irregular surface is typically characterized using mean roughness [43]. Figure 1 shows topographical images of the superhydrophobic nanocomposite, and Table 2 provides the calculated parameters. The average roughness of all surfaces was less than 1.2 µm, and the arithmetic mean roughness was around 156 nm. The roughness increased with higher concentrations of nanosilica. According to the study by Davis et al. [44], the superhydrophobic coatings exhibited a roughness value of approximately 1.6 µm, which contributed to the robustness of their surfaces due to this level of smoothness. In contrast, our experiments, which achieved an average roughness of 132 nm, position our tested surfaces among the smoothest superhydrophobic nanocomposites applied through the spraying technique. Water droplets effortlessly rolled across the superhydrophobic surface of the prepared coatings, indicating that the Cassie model better explained our observations [45,46]. The Cassie equation is given as

cos 𝜃_𝐴_ = 𝑓_1_cos 𝜃 − 𝑓_2_cos *θ_A_* − *f*_1_cos *θ* − *f*_2_,

where *θ_A_* is the measured apparent CA on the superhydrophobic coated surface, *θ* is the WCA on a polyurethane-coated surface (measured at 91°), the terms *f*_1_ and *f*_2_ represent the fractions of solids and air in contact with water droplets, respectively, and they satisfy *f*_1_ + *f*_2_ = 1. For an average WCA of 164°, *f*_1_ was 3.9%, 3.4%, and 1.87% for FPU-SiO_2_ 4%, FPU-SiO_2_ 7%, and FPU-SiO_2_ 10% samples, respectively, indicating that 96.1%, 96.6%, and 98.13%, respectively, of their surfaces were occupied by air. These results demonstrate that the combination of silica nanoparticles and polyurethane allows air to be easily trapped to create a highly superhydrophobic surface.

### 2.2. Ice Nucleation Temperature and Freezing Time Delay

**Figure 2:**
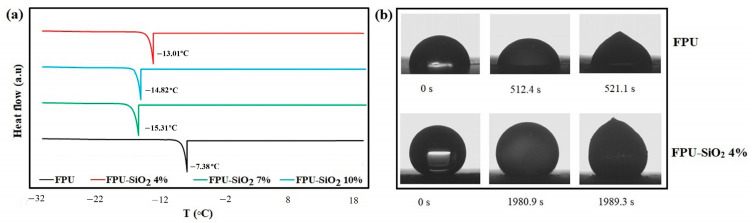
(**a**) Evaluation by differential scanning calorimetry (DSC) of ice nucleation temperatures; (**b**) freezing delay time for the FPU and FPU-SiO_2_ 4% coatings.

Using differential scanning calorimetry (DSC), the durations needed for the initiation of ice nucleation in a water droplet placed on the various coatings were measured. Silica nanoparticles lowered the freezing point (Figure 2a). Previous studies have indicated that maintaining a surface roughness of less than 35 nm is necessary for homogeneous ice nucleation. At this roughness level, the nucleation temperature decreases to −38 °C. On the other hand, the nanoparticles serve as sites for ice nucleation, causing heterogeneous nucleation [47,48]. The 4 wt.% nanoparticle concentration led to a 4 °C decrease in the freezing temperature because the greater nanometric roughness of the coating favored more trapped air. As the nanoparticle concentration increased, the freezing temperature was higher because of the increase in ice nucleation sites caused by nanoparticle accumulation.

Because the 4 wt.% sample produced the greatest decrease in ice nucleation time, presented a crack-free surface, and offered suitable surface morphology, we selected this nanosilica concentration for all subsequent tests of the polyurethane nanocomposite coating.

Using a superhydrophobic coating significantly reduces the ice nucleation rate and the speed of ice growth because of the reduced solid–liquid contact area caused by trapped air pockets [10]. For instance, reducing the solid–liquid contact area to 3.9% for FPU-SiO_2_ 4% delayed the nucleation process on the surface because trapped air in the surface roughness limits heat exchange between the solid and liquid surfaces. The freezing experiments with water droplets shown in Figure 2b indicate that it takes 1989 s for ice to form on the prepared superhydrophobic surface, whereas it takes 521 s for a coating that lacks nanoparticles. Previous studies have recorded ice formation times on commercial silicone samples that range from 200 to 1500 s, depending on the temperature of the freezing chamber [10,49]. Our experiments on the superhydrophobic sample, therefore, demonstrated a significant delay in ice formation time.

### 2.3. Ice Adhesion

**Figure 3:**
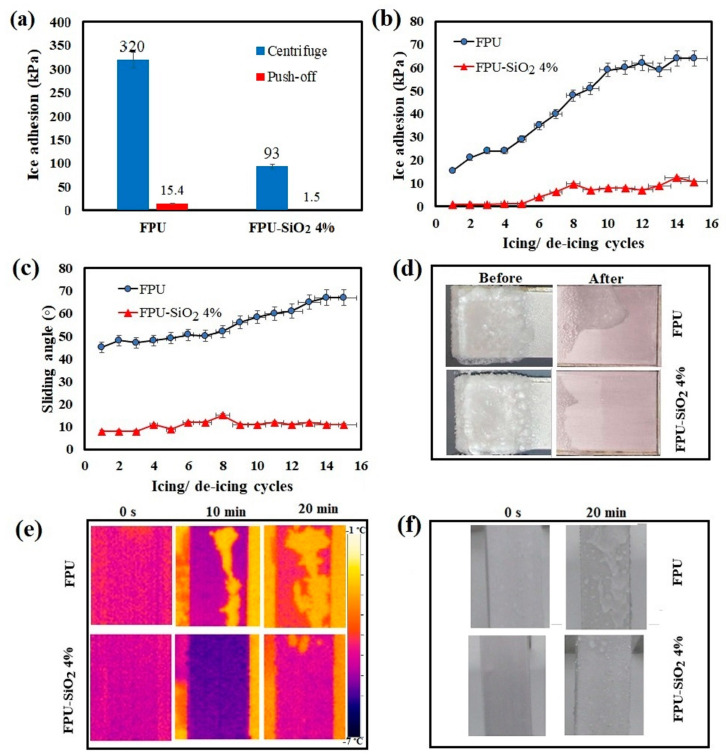
(**a**) Ice adhesion strength measurements determined using push-off and centrifuge tests for the FPU and FPU-SiO_2_ 4% samples; (**b**) ice adhesion strength; (**c**) sliding angle of the FPU and FPU-SiO_2_ 4% coatings over 15 icing/de-icing cycles; (**d**) optical images of the FPU and FPU-SiO_2_ 4% samples before and after the centrifuge test; (**e**) infrared thermographs of the FPU and FPU-SiO_2_ 4% samples during 20 min of precipitation at an incline of 80°; (**f**) optical images of the FPU and FPU-SiO_2_ 4% samples after 20 min of precipitation at an incline of 80°.

The adhesive strength of the ice/substrate is another important parameter for evaluating icephobicity. A lower adhesive strength allows the ice to detach more easily from the surface. In this study, we evaluated both non-impact and impact ice, given the various ice forms used in previous research on ice adhesion. We ran a push-off test (Figure 3a) to evaluate the adhesion strength of non-impact ice on coating surfaces. For testing impact ice, we sprayed supercooled water droplets onto the coated surface at subzero temperatures at a controlled velocity and volume and then subjected the frozen sample to a centrifuge test. This latter method subjects the surface to harsher ice conditions than the push-off test. The presence of PDMS-modified nanosilica particles reduced ice adhesion from 430 kPa (for FPU) to 90 kPa (FPU-SiO_2_ 4%) (Figure 3a). The low ice adhesion for the FPU-SiO_2_ 4% samples can be attributed to the lower contact area between the ice and coating surface because of the air trapped on the coating [42]. The greater ice adhesion value for the FPU-lacking nanoparticles confirms how the superhydrophobic surface of the FPU-SiO_2_ 4% sample also offers icephobicity. For the harsher impact icing conditions, the shear stresses between the coated samples and ice were considerably less than those recorded between the ice and the Al substrates. A surface can be considered icephobic when the adhesion strength of non-impact ice is less than 100 kPa [10]. However, as the conditions for impact ice are harsher, the adhesion strength threshold could be deemed higher. Regardless, the FPU-SiO_2_ 4% produced a shear stress of less than 100 kPa, even for impact icing.

Although topological factors are the main elements that directly influence ice adhesion on superhydrophobic surfaces—because of the air trapped at the surface within the roughness features—the durability of these developed coatings remains essential for its application in real-world settings. Therefore, we tested ice adhesion via push-off tests over 15 icing/de-icing cycles. The ice adhesion strength of the coatings gradually increased with each icing/de-icing cycle because of a gradual loss of roughness (Figure 3b). This phenomenon has been previously reported for anti-icing surfaces/coatings produced using silica nanoparticles [20].

After 15 cycles, the ice adhesion of the FPU-SiO_2_ 4% sample remained below 10 kPa, significantly lower than that of the FPU coating (64 kPa). An ice adhesion strength of less than 11 kPa over 15 cycles indicates that the coating is a good candidate for passive ice removal [50], as this value is lower than natural forces that can remove ice, such as gravity, vibration, and wind; thus, any accumulated ice can be easily detached [51]. The FPU-SiO_2_ 4% sample had the lowest SA (10°) (Figure 3c), confirming the effective role of roughness and the low surface energy of silica nanoparticles on the coating’s uppermost layer for enhancing wetting behavior and icephobic performance [20]. Optical images of the sample surfaces before and after the centrifuge test clearly show a much lower area of ice on the surface of the FPU-SiO_2_ 4% sample than on the FPU surface (Figure 3d).

We then ran an ice accretion test to illustrate the icephobic characteristics of the coatings. Infrared thermographs were taken of the samples at an incline of 80° and exposed to 20 min of precipitation to investigate the role of silica nanoparticles in heat transfer during ice accretion. We observed that the initial temperature of the samples was approximately −3.9 °C (Figure 3e). After 20 min of exposure, the surface temperatures of the FPU and FPU-SiO_2_ 4% samples were −3.5 and −3.1 °C, respectively, confirming the positive effect of silica nanoparticles on overall heat insulation. The ice formed on the samples after 20 min of exposure is illustrated in Figure 3f. The FPU-SiO_2_ 4% sample had a lower ice accretion (0.64 g) than the FPU sample (4.32 g) at an 80° inclination. The ice formed on the FPU sample occurred in a Runback manner, which occurs when supercooled water hits the surface and flows over it to then freeze. Conversely, the ice formed on the superhydrophobic sample was only observed at two isolated points, which resemble islands. Mangini et al. [52] reported similar types of ice formations, including Runback and island formations, on hydrophilic and superhydrophobic surfaces, respectively.

For a final series of experiments, we applied the FPU-SiO_2_ 4% nanocomposite coating to a Ti/Ce-CC-modified aluminum surface to enhance the corrosion protection and adhesion strength of the organic layer applied to the aluminum substrate. We tested the prepared surfaces for corrosion resistance, adhesion, and changes in CA during simulated weathering.

### 2.4. Performance of a Polyurethane Nanocomposite When Coupled with a Titanium-Based Conversion Coating

#### 2.4.1. Anticorrosion Performance and Coating Adhesion

**Figure 4:**
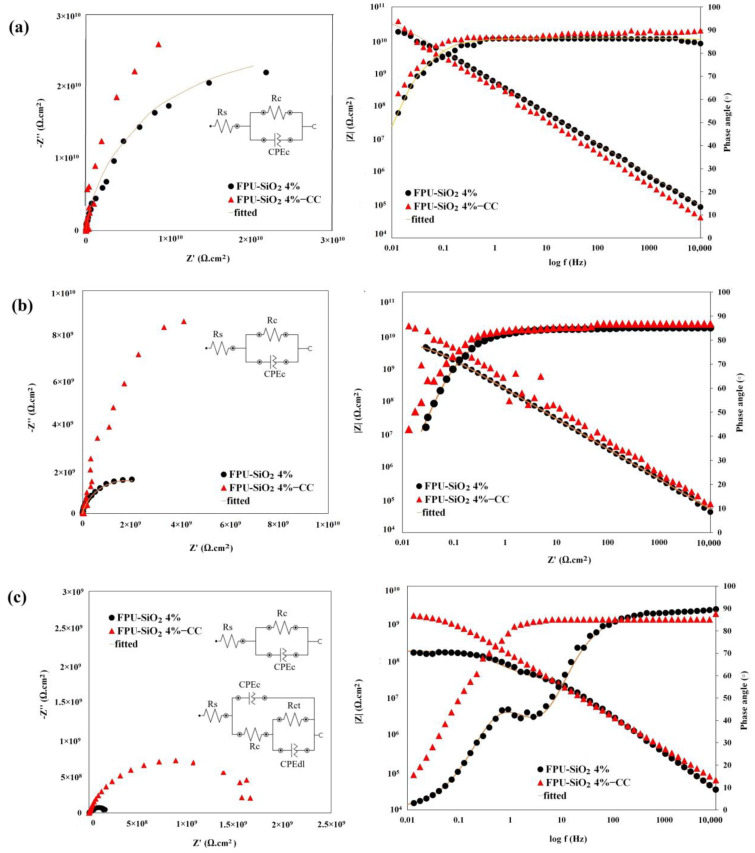
Nyquist and Bode diagrams of FPU-SiO_2_ 4% and FPU-SiO_2_ 4%-CC samples immersed in a NaCl 3.5 wt.% solution (**a**) after 1 day; (**b**) after 60 days; (**c**) after 90 days.

EIS assessed the electrochemical properties of coated samples subjected to a 3.5 wt.% NaCl solution, and the results are visually represented in Nyquist and Bode diagrams (Figure 4). Various electrical equivalent circuits (EECs) were applied to analyze the experimental data (Table 3). R_s_, R_c_, and R_ct_ represent the solution resistance, coating resistance, and charge transfer resistance, respectively. CPE_c_ and CPE_dl_ represent the constant phase elements of the coating and the double layer, respectively. All coating systems initially exhibited very high resistance (R_c_) with a single capacitive loop (Figure 4a). The Bode plots show a single time constant and a straight line with a slope of −1 (Figure 4a), indicating the strong barrier property of the coatings. We therefore used the EEC model in Figure 4a to fit the EIS data. The impedance measurements in the Bode diagrams for all the samples show values greater than 10^9^ Ω·cm^2^ at low frequencies (0.01 Hz), testifying to the effectiveness of the barrier coatings [41]. In Bode plots, the impedance at low frequencies reflects the developed coating’s barrier properties, making it suitable for assessing the comparative anticorrosion effectiveness of the coatings [53,54,55]. After eight weeks of immersion in NaCl 3.5 wt.% (Figure 4b), the coatings maintained their high resistance and exhibited capacitive behavior. A noticeable difference in corrosion resistance between FPUSiO_2_ 4%-CC and FPUSiO_2_ 4% was observed when the immersion time was increased to 90 days (Figure 4c), highlighting the role of the conversion coating in significantly increasing the impedance value at low frequencies (about 10 times) through its barrier effect. The superhydrophobic nature of these coatings delayed the penetration of corrosive agents; after 90 days of immersion, the conversion coating’s role was evident. The FPUSiO_2_ 4%-CC sample offered adequate corrosion resistance via three means. The first occurs at the interface of the aluminum substrate and the organic coating, involving the titanium–cerium conversion coating. This coating alters the physical and chemical properties of the surfaces to create favorable conditions for stronger mechanical–physical interactions with the organic coating. Moreover, assuming the penetration of the corrosive electrolyte through the organic layers to the metal, the conversion coating limits the access of corrosive ions to the metal surfaces, thus increasing the charge transfer resistance because of the presence of the conversion coating on the metal and effectively protecting the aluminum substrate. A study of a polyurethane-based coating system on an aluminum surface modified with a titanium-based conversion coating concluded that the increased adhesion due to the presence of the conversion coating at the metal–organic interface was crucial for preventing the coating from detaching from the metal [41].

**Figure 5:**
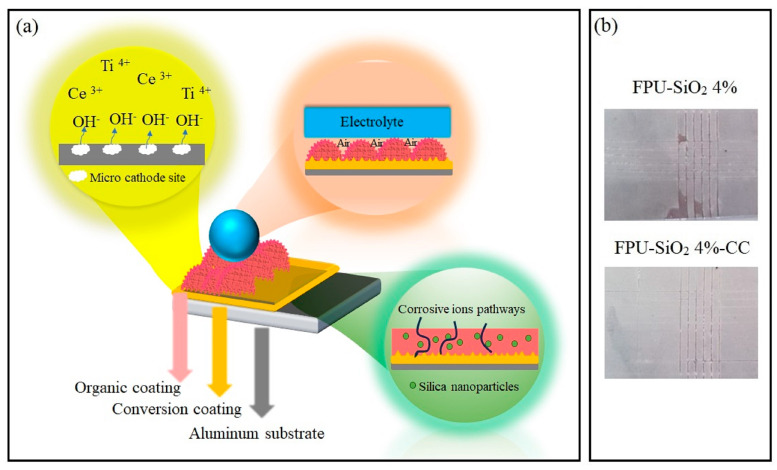
(**a**) Schematic of the proposed mechanism of corrosion protection of FPU-SiO_2_ 4%-CC; (**b**) appearance of the FPU-SiO_2_ 4% and FPU-SiO_2_ 4%-CC coating surfaces after the cross-cut test.

The second means involves the dispersion of nanosilica particles. Well-dispersed nanoparticles can significantly delay the penetration of the electrolyte into the metal surface by postponing the initial contact and consequently delaying the onset of corrosion reactions [12,56]. The final means involves incorporating nanosilica into the polyurethane matrix to create appropriate surface roughness for producing superhydrophobic coatings and repelling aqueous electrolytes from the surface [20]. Figure 5a shows the schematic of the proposed mechanisms of corrosion protection of the FPU-SiO_2_ 4% sample.

The Bode diagrams show that the FPUSiO_2_ 4%-CC sample resists corrosion even after 90 days of immersion and that no break frequency can be determined. After 1, 60, and 90 days of immersion in a corrosive electrolyte, the FPUSiO_2_ 4%-CC sample retained an impedance modulus of around 10^9^ (Table 3), confirming that the coating continues to protect the surface very well after 90 days in a corrosive environment. 

To gain a better understanding of the titanium/cerium conversion coating’s performance, the mechanism of its formation is described below [41]. 

To begin the film formation reactions of the conversion coating on the Al-2024 substrate, it is necessary to remove the native oxide layer. This is achieved by immersing Al-2024 in the conversion solution, where fluoride ions, produced from the dissolution of H_2_TiF_6_, dissolve the oxide layer, thereby enabling the anodic reactions to commence as follows:

Al_2_O_3_ + 6H^+^ + 6F^−^ → 2AlF_3_ + 3H_2_O (1)

Al → Al^3+^ + 3e^−^ (2)

Al^3+^ + TiF_6_^2−^ → AlF_6_^3−^ + Ti^4+^. (3)

Generally, an elevation in local pH is observed as a result of the following cathodic reactions:

O_2_ + 4H_2_O + 4e^−^ → 4OH^−^ (4)

2H^+^ + 2e^−^ → H_2_. (5)

The formation of the coating requires the presence of hydroxyl ions. The formation of the coating’s film proceeded according to the reactions detailed below:

TiF_6_^2−^ + 4OH^−^ → TiO_2_⋅2H_2_O(s) + 6F^−^ (6)

or

TiF_6_^2−^ + 4H_2_O → TiO_2_⋅H_2_O(s) + 6HF + O^2−^. (7)

In the presence of cerium, the probable interactions between cerium ions, hydroxyl ions, and the water solution are illustrated by the following reactions:

Ce^3+^ + 3OH^−^ → Ce(OH)_3,_ (8)

Ce^3+^ + H_2_O → Ce(OH)_2_^2+^ + 2H^+^ + e^−^_,_ (9)

Ce(OH)_2_^2+^ + 2OH^−^ → CeO_2_⋅2H_2_O_,_ (10)

and

CeO_2_⋅2H_2_O → CeO_2_ + 2H_2_O. (11)

Given the reactions outlined above, it is likely that the oxide and hydroxide forms of Ce and Ti are incorporated into the coating structure. For this reason, the Ti/Ce-CC can enhance the corrosion resistance of the system by blocking cathodic areas and promoting uniform coating growth across the metal substrate. 

To evaluate the effect of the conversion coating on the adhesion of the organic layer to the substrate, we performed a cross-cut test on the samples after they were immersed for two weeks in distilled water. Figure 5b shows that the adhesion of the coating applied to the modified aluminum with the conversion coating was approximately 5B, whereas the FPU-SiO_2_ 4% sample had detached in some areas. Therefore, the conversion coating is recommended to enhance adhesion. Conversion coatings improve the adhesion strength through mechanical interlocking and wetting mechanisms. As shown in our recent study, the increase in the roughness parameter improves adhesion through a mechanical interlocking mechanism, and the wetting mechanism alters the adhesion strength through a change in surface chemistry [41]. 

In the current study, the substrate also played an important role. The titanium oxide/hydroxide present on the Al substrate promoted Bronsted interactions with the polar groups of the organic matrix [40]. Moreover, the presence of oxide and hydroxide forms of metals enhanced the attraction between the substrate and polar groups of the organic coating [57].

#### 2.4.2. Weathering Resistance

**Figure 6:**
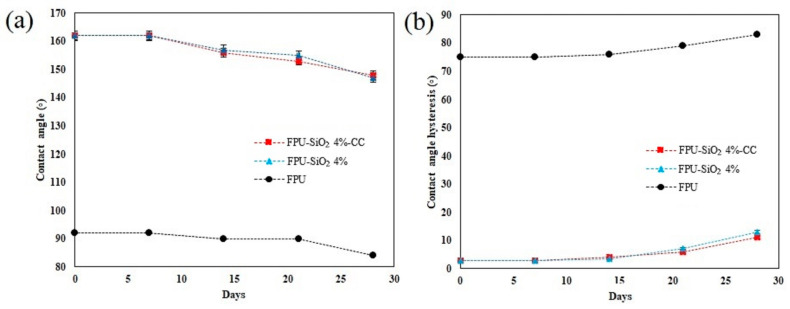
(**a**) Contact angle and (**b**) contact angle hysteresis of the FPU, FPU-SiO_2_ 4%, and FPU-SiO_2_ 4%-CC over 28 days of exposure during a weathering test.

The CA and CAH of the FPU, FPU-SiO_2_ 4%, and FPU-SiO_2_ 4%-CC samples after 28 days of weathering exposure are shown in Figure 6. Both the PU-SiO_2_ 4% and FPU-SiO_2_ 4%-CC coatings maintained their superhydrophobic properties for up to 21 days; however, their respective CAHs after 28 days were approximately 11.2° and 12.9°, reflecting a loss of superhydrophobicity. This loss may stem from nanoparticles detaching from the matrix, leading to the degradation of the hierarchical structure on the coating. The weathering tests also affected the visual properties of the coatings. Table 4 lists the differences in L, a, and b values before and after the QUV test, which quantify the color changes induced by exposure. The polyurethane-based coatings exhibited the most significant color changes, whereas those containing water-repellent silica nanoparticles showed smaller color changes (i.e., smaller ΔE values). The coatings with water-repellent nanosilica thus experienced less degradation than the polyurethane coatings alone. The difference in ΔE values between the FPU-SiO_2_ 4% and FPU-SiO_2_ 4%-CC samples may be attributed to corrosion within the coatings. Moradian et al. demonstrated that the presence of water-repellent silica nanoparticles enhances the weather resistance of polyurethane coatings [58].

## 3. Materials and Methods

For the substrate, we selected aluminum 2024T3 (AA2024T3) sheets comprising (in wt.%) 4.1% of Cu, 1.5% of Mg, 0.7% of Mn, 0.5% of Fe, 0.3% of Zn, 0.15% of Ti, 0.1% of Ni, and the remainder of Al. The AGC Chemical Company (Exton, Pennsylvania, USA) provided the fluoropolyol (solid content = 66% ± 1%, hydroxyl content = 100). Polyisocyanate resin with an NCO content of 16.5% was supplied by the Bayer Company (Del Mar, California, USA), and hydrophobic fumed silica (<20 nm particle size) was purchased from Evonik Industries (Essen, Germany). Nitric acid was purchased from the Fisher scientific Company (Ottawa, Ontario, Canada). Sigma Aldrich (Oakville, Ontario, Canada) provided the H_2_TiF_6_ solution (60 wt.%), whereas the Merck Company (Rahway, NJ, USA) supplied the cerium nitrate, which was used as an additive. The optimal conditions for preparing the titanium/cerium conversion coating were 1 g/L of H_2_TiF_6_ solution (60 wt.%), 0.5 g/L of cerium nitrate solution, a pH of 4.5, an immersion time of 2 min, and an ambient temperature of 38 °C.

To prepare the nanocomposites, we mixed various amounts of nanoparticles (4, 7, and 10 wt.% based on the weight of the resin and hardener) with methyl ethyl ketone (MEK). The mixture was sonicated at 200 W for 10 min to ensure the uniform dispersion of the silica nanoparticles. The solvent–nanoparticle blend was then combined with a polyurethane-based resin and sonicated again at 200 W for 3 min. An ice bath was used during sonication to maintain a low temperature. The hardener was then gently mixed into the suspension for 5 min. Additional MEK was added as necessary to adjust the viscosity of the final mixture. The resulting mixtures were sprayed onto a pretreated AA2024-T3 substrate using air pressure of 4 bar and a spray distance of 25 cm. Using a relatively higher pressure and greater spray distance promoted finer droplets and, therefore, faster solvent evaporation, and it reduced the likelihood of deforming the wet film during spraying. The coatings were left to cure at room temperature for one week. The coating’s final dry thickness was 12 ± 2 µm. The sample codes used were FPU for coatings without nanoparticles, FPU-SiO_2_ 4%, FPU-SiO_2_ 7%, and PU-SiO_2_ 10% for coatings with 4, 7, and 10 wt.% silica nanoparticles, respectively. FPU-SiO_2_ 4%-CC represented the coating containing 4 wt.% hydrophobic silica particles applied to a pretreated substrate with the titanium/cerium conversion coating [20,41].

We studied the effect of embedded nanosilica on the surface of the polyurethane coatings using atomic force microscopy (AMBIOS Tech, Santa Cruz, California). The impact of various concentrations of these nanoparticles on ice formation on FPU coatings was assessed using differential scanning calorimetry (DSC). We placed 5 mg of water in a DSC pan coated with a thin layer of each coating and covered it with a lid, using an empty pan as a reference. The procedure involved cooling it from +20 to −30 °C at a rate of 5 °C per min. Additionally, we used a Kruss contact angle meter with the cooling chamber temperature maintained at −20 °C to explore how different concentrations of SiO_2_ nanoparticles influenced the freezing delay time of the FPU sample.

The adhesive strength of ice on test samples was assessed through push-off and centrifuge tests. In the push-off test conducted at −10 °C, a plastic mold with a diameter of 1.2 cm was placed on the samples and filled with deionized water. After freezing the samples for 24 h, we measured the force required to detach the ice using a force gauge to determine ice adhesion. For the centrifuge test, glaze ice was created by spraying water cooled to −8 °C onto the sample surface to produce a 6 ± 0.5 mm thick ice layer for 35 min. After an hour, the ice adhesion strength was tested using a centrifuge in a cold room set at −10 ± 0.2 °C. The force needed to dislodge the ice from the samples at the end of a beam was recorded, and the adhesion strength was calculated by dividing this force by the area covered with ice. The adhesion reduction factor was

$$ARF=\frac{\tau_{Al}}{\tau_{coating}}\;,$$

where $\tau_{Al}$ and $\tau_{coating}$ represent the shear stress of the aluminum substrate and samples, respectively.

We measured WCA using a Kruss™ DSA 100 goniometer (KRUSS Scientific, Chaussee, Germany) at both room temperature and subzero conditions. For the static CA tests, we deposited a 4 µL droplet of distilled water onto the coated surfaces, and the angle was determined through drop shape analysis using the Young–Laplace approximation method. The results are presented as the mean of five random measurements taken from each sample’s surface, along with the standard deviation.

We assessed the ice buildup on the prepared coatings using the static accumulation test (SAT). Coated aluminum substrates measuring 3 cm × 15 cm were positioned on a sample holder at angles of 80° and maintained at −5 °C. The samples were then subjected to precipitation from microdroplets (average diameter of 327 µm) at 4 °C for 20 min. After cooling the samples at −5 °C for 45 min, we measured the ice accumulation of the mass of accreted ice. The surface temperature of the samples was tracked using an Optris PIX infrared camera (Optris, Berlin, Germany).

Electrochemical impedance spectroscopy (EIS) was conducted using an Autolab 302N (Autolab, Metrohm, The Netherlands). A standard three-electrode cell setup was used, with the Ag/AgCl electrode and a platinum rod serving as the reference and counter electrodes, respectively. The experiments involved a 3.5 wt.% NaCl solution as the electrolyte, and each sample had an exposed area (with a diameter of 1.5 cm). Prior to each test, the samples were immersed in a 3.5 wt.% NaCl solution to stabilize their potential values. The frequency for the EIS measurements ranged from 10 mHz to 10 kHz.

The adhesion properties of the developed nanocomposites were assessed using the cross-cut adhesion tape test, following ASTM 3359B [59]. Weathering tests were conducted according to the ASTM G154 standard [60]. UV-A fluorescent lamps with a peak of 340 nm from QUV (Q-Lab Corporation, Westlake, OH, USA) were used in the chamber. Coated films on aluminum substrates were exposed to ultraviolet radiation at 62 °C for 1 h, followed by 2 h of water condensation at 50 °C at an irradiance of 0.89 W·m^−2^.

## 4. Conclusions

This study demonstrates how polyurethane nanocomposite coatings enhanced with nanosilica and titanium-based conversion coatings produce excellent anticorrosion and icephobic protection for exposed surfaces. We developed thin icephobic nanocomposite coatings using a simple method and varying concentrations of hydrophobic silica nanoparticles. We analyzed the coatings using CA and CAH measurements, FE-SEM, and AFM techniques. Increasing the concentration of silica nanoparticles heightened the static contact angle of water on the surface from 92° to 167°, with the lowest contact angle hysteresis (4°) observed with the 4 wt.% nanosilica coating. Higher nanoparticle concentrations increased surface roughness from 28 nm to 283 nm, likely because of nanoparticle aggregation.

We evaluated the adhesion of non-impact and impact ice to the coating using push-off and centrifuge tests on FPU-SiO_2_ 4% and observed much greater icephobicity (ARF of 4.89 and 3.88 for the push-off and centrifuge tests, respectively) than that of the FPU sample. After 15 icing/de-icing cycles, the ice adhesion strength remained below 20 kPa, demonstrating the coating’s durability with respect to icing events. Moreover, the coatings significantly delayed the onset of ice formation from 521 s (for the FPU) to 1989.3 s (for FPU-SiO_2_ 4%) at −20 °C.

We improved the durability of these coatings by applying a titanium/cerium-based conversion coating to the aluminum substrate. We assessed the protective properties of the coatings using EIS, and the 4% nanosilica coating on the titanium-based conversion coating provided the highest surface protection when the samples were placed in a 3.5 wt.% NaCl solution, as evidenced by an impedance value of 1.6 × 10^9^ Ω·cm^2^, even after 12 weeks of immersion. A cross-cut adhesion test demonstrated a strong adhesion of the 4 wt.% nanosilica coating when coupled with the titanium/cerium–based conversion coating. Therefore, we found that the polyurethane nanocomposite coating having a 4 wt.% silica nanoparticle concentration and coupled with a titanium/cerium-based conversion coating achieved an optimal balance of icephobicity, anticorrosion performance, and adhesion strength.

## Figures and Tables

**Table 1:** Contact angle and contact angle hysteresis values of FPU, FPU-SiO_2_ 4%, FPU-SiO_2_ 7%, and FPU-SiO_2_ 10% samples.

Sample Code		Contact Angle (°)	Contact Angle Hysteresis (°)
FPU	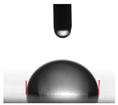	91 ± 1	75 ± 3
FPU-SiO_2_ 4%	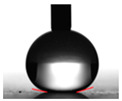	164 ± 3	3 ± 0.5
FPU-SiO_2_ 7%	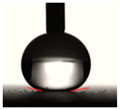	165 ± 2	9 ± 1
FPU-SiO_2_ 10%	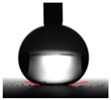	169 ± 3	25 ± 1

**Table 2:** The roughness of the FPU, FPU-SiO_2_ 4%, FPU-SiO_2_ 7%, and FPU-SiO_2_ 10% coatings.

Coating	Average Roughness (nm)
PU	28.2 ± 7.1
PU-SiO_2_ 4%	132.1 ± 9.5
PU-SiO_2_ 7%	201.3 ± 11.3
PU-SiO_2_ 10%	283.1 ± 8.1

**Table 3:** Electrochemical impedance values obtained from electrical equivalent circuits (EEC) for FPU-SiO_2_ 4% and FPU-SiO_2_ 4%-CC samples after 1 day, 60 days, and 90 days of immersion in a 3.5 wt.% NaCl solution.

Sample Code	|Z|@0.01 Hz (Ω·cm^2^) 1 Day	|Z|@0.01 Hz (Ω·cm^2^) 60 Days	|Z|@0.01 Hz (Ω·cm^2^) 90 Days
FPU-SiO_2_ 4%	1.7 × 10^10^	2.2 × 10^9^	1.6 × 10^8^
FPUSiO_2_ 4%-CC	3 × 10^10^	9 × 10^9^	1.6 × 10^9^

**Table 4:** Parameters values of ΔL*, Δa*, Δb*, and ΔE* of the FPU, FPU-SiO_2_ 4%, and FPU-SiO_2_ 4%-CC during 28 days of exposure during a weathering test.

Sample Code	ΔL*	Δa*	Δb*	ΔE*
FPU	0.74	−1.00	3.05	3.3
FPU-SiO_2_ 4%	−0.13	−1.07	1.05	2.1
FPU-SiO_2_ 4%-CC	−0.75	−0.95	1.59	1.9

## Data Availability

The original contributions presented in the study are included in the article, further inquiries can be directed to the corresponding authors.
